# Screening colonoscopy and flexible sigmoidoscopy for reduction of colorectal cancer incidence: A case-control study

**DOI:** 10.1371/journal.pone.0226027

**Published:** 2019-12-05

**Authors:** Cynthia W. Ko, V. Paul Doria-Rose, Michael J. Barrett, Aruna Kamineni, Lindsey Enewold, Noel S. Weiss

**Affiliations:** 1 Division of Gastroenterology, Department of Medicine, University of Washington School of Medicine, Seattle, Washington, United States of America; 2 National Cancer Institute, National Institutes of Health, Bethesda, Maryland, United States of America; 3 Information Management Services, Inc., Calverton, Maryland, United States of America; 4 Kaiser Permanente Washington Health Research Institute, Seattle, Washington, United States of America; 5 Department of Epidemiology, University of Washington, Seattle, Washington, United States of America; University of Munich, GERMANY

## Abstract

**Background:**

Flexible sigmoidoscopy and colonoscopy are both recommended colorectal cancer screening options, but their relative effectiveness needs clarification. The aim of this study was to compare the effectiveness of colonoscopy and flexible sigmoidoscopy for reduction of colorectal cancer incidence.

**Methods:**

We conducted a case-control study within the linked Surveillance, Epidemiology, and End Results (SEER)-Medicare database. Cases were subjects age 70–85 years in the SEER-Medicare database diagnosed with CRC during 2004–2013. Up to 3 controls were matched to each case by birth year, sex, race, and SEER region. Receipt of screening colonoscopy or flexible sigmoidoscopy was ascertained from Medicare claims. Conditional logistic regression models were developed to estimate the odds ratios (ORs) and 95% confidence intervals (CI) for a history of screening in cases vs. controls. We conducted secondary analyses by sex, race, endoscopist characteristics, and with varying timing and duration of the look-back period.

**Results:**

Receipt of screening colonoscopy and sigmoidoscopy was associated with a 59% (OR 0.41, 95%CI 0.39, 0.43) and 22% reduction (OR 0.78, 95%CI 0.67, 0.92) in colorectal cancer incidence, respectively. Colonoscopy was associated with greater reduction in the distal colorectal cancer incidence (OR 0.22, 95%CI 0.20, 0.24) than proximal colorectal cancer incidence (OR 0.62, 95%CI 0.59, 0.66). Sigmoidoscopy was associated with a 52% reduction in distal colorectal cancer incidence (OR 0.48, 95%CI 0.37, 0.63), but with no reduction in proximal colorectal cancer incidence. These associations were stronger in men than in women. No differences by race or endoscopist characteristics were observed.

**Conclusion:**

Both screening colonoscopy and sigmoidoscopy were associated with reductions in overall colorectal cancer incidence, with a greater magnitude of reduction observed with colonoscopy.

## Introduction

Screening for colorectal cancer (CRC) for average risk individuals is widely recommended [1–3], with potential options including colonoscopy, flexible sigmoidoscopy with or without fecal immunochemical testing (FIT), or annual FIT alone. Fecal occult blood testing (FOBT) and flexible sigmoidoscopy reduce overall CRC incidence and mortality in randomized controlled trials [4–12], but only observational evidence supporting screening colonoscopy is currently available [13–18]. Although randomized controlled trials of screening colonoscopy are in progress [19–22], results are not expected until 2022 [23] or 2026–7 [21,22].

Colonoscopy is currently the dominant screening modality in the United States due to its perceived superiority for detecting and removing polyps throughout the colon and rectum [24–27], potentially preventing their malignant transformation and reducing CRC incidence. However, flexible sigmoidoscopy has several potential advantages over colonoscopy, including lower complication risk [28], less need for sedation, less intensive endoscopist training [29,30], and lower cost. Randomized controlled trials have demonstrated that flexible sigmoidoscopy is less effective in reducing CRC incidence and mortality in the proximal colon than in the distal colorectum [10,11,31,32], but observational studies suggest similar differences for colonoscopy [15–18,33,34]. These observational studies have methodologic limitations, primarily related to insufficient information about colonoscopy indication, which may impact their validity [35,36].

Comparisons of screening colonoscopy and sigmoidoscopy in the same underlying population are lacking, and understanding their comparative effectiveness is crucial for making rational screening choices [37–39]. The aim of this study was to compare the effectiveness of flexible sigmoidoscopy and colonoscopy in reducing CRC incidence.

## Materials and methods

The study was based in the Surveillance, Epidemiology, and End Results (SEER)-Medicare database, which links administrative records of the Medicare program with cancer surveillance data [40].

### Case identification and control selection

Cases were identified from the SEER-Medicare Patient Entitlement and Diagnosis Summary File (PEDSF), and were subjects age 70 to 85 years with CRC diagnosed from 2004–2013 as their first malignancy. Age 70 was chosen as the minimal diagnosis age to allow for at least 5 years in which to assess screening histories (i.e. the look-back period) as Medicare enrollment most commonly begins at age 65. To encompass time periods after the introduction of Medicare CRC screening coverage with flexible sigmoidoscopy in 1999 and with colonoscopy for average-risk individuals in mid-2001 and to allow sufficient elapsed time for screening to reduce CRC incidence, we did not include cases diagnosed before 2004. To maximize completeness of claims submission, we required continuous enrollment in both Part A and Part B fee-for-service Medicare without health maintenance organization enrollment for at least 5 years prior to the diagnosis date. Part A Medicare covers inpatient hospital services, while Part B covers outpatient medical services, including preventive care. Enrollment in both Part A and Part B ensures that all medical services received can be accurately ascertained. Cases with an International Classification of Disease-9-Clinical Modification (ICD-9-CM) code indicating a prior CRC (V10.05, V10.06) or inflammatory bowel disease diagnosis (555.x, 556.x) or a barium enema or computed tomography colonography more than 6 months before the diagnosis date, or evidence of a colorectal resection at any time before diagnosis were excluded.

Controls were identified from the 5% non-cancer SEER-Medicare sample [40]. Controls had the same inclusion and exclusion criteria as cases and were required to have an available look-back period at least as long as their matched cases. Controls and cases were matched by calendar year of birth, sex, race, and SEER region on the reference date (date of matched case’s CRC diagnosis) and had to have been alive at the reference date. We randomly selected up to 3 controls from the eligible pool per case. Cases with no available controls were excluded.

### Ascertainment of colonoscopy and flexible sigmoidoscopy

We identified receipt of colonoscopy or flexible sigmoidoscopy from the Carrier, Outpatient, and MedPAR (inpatient) files in the Medicare claims data using International Classification of Disease-9-Procedures (ICD-9-P), Current Procedural Terminology (CPT) and Healthcare Common Procedure Coding System (HCPCS) codes (S1 Table). As the clinical indication for endoscopy is an important consideration, we used a previously published algorithm to classify the colonoscopy indication as screening, surveillance, or diagnostic [41]. This algorithm incorporates ICD-9-CM diagnosis and CPT/HCPCS procedure codes from the Medicare colonoscopy claim and claims in the 12 months prior, demographic information, and site of colonoscopy service. The sensitivity and specificity of this algorithm for a diagnostic colonoscopy indication are 76% and 83%, respectively, with an overall accuracy of 72%. We adapted this algorithm to identify indications for sigmoidoscopy, as we expected similar coding patterns [42]. Because the vast majority of inpatient colonoscopies and sigmoidoscopies were performed for diagnostic indications, inpatient exams were always classified as diagnostic. Endoscopies classified as screening or surveillance by this algorithm were retained and tabulated as exposures for the analysis. Receipt of diagnostic procedures was used to determine if a cancer was clinically-detected or screen-detected; however, in our analyses, diagnostic procedures were not counted as screening exams.

### Definition of the look-back period for ascertainment of CRC screening histories

**Fig 1:**
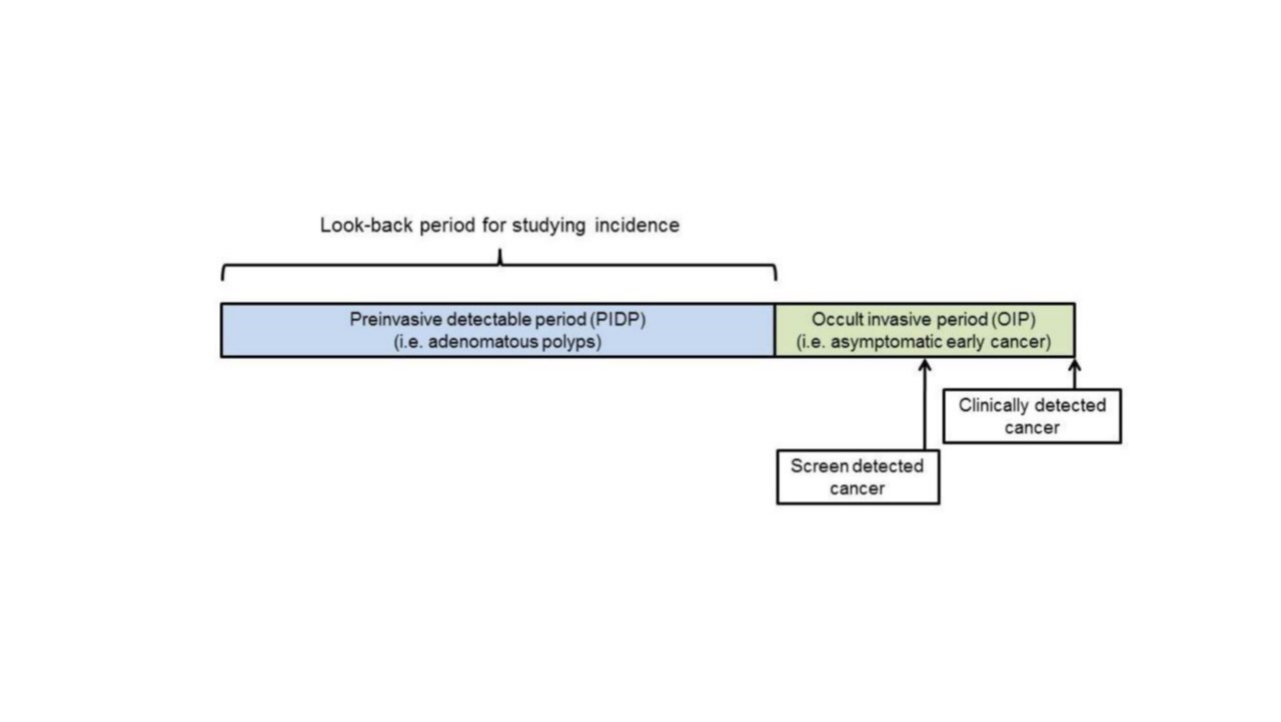
Look-back periods for evaluating screening and colorectal cancer incidence.

Due to SEER-Medicare files linkage protocol constraints, eligible subjects had claims available for 5 to 7 years prior to the reference date [43]. In case-control studies of cancer incidence and screening, the goal is to evaluate procedures occurring in the pre-invasive detectable phase (PIDP), but prior to the occult invasive phase (OIP) where an asymptomatic invasive cancer is already present (Fig 1) [44]. For CRC, the PIDP corresponds to the period where adenomatous or sessile serrated polyps are present but have not undergone malignant transformation. The PIDP cannot be directly observed but is commonly estimated at up to 10 years for CRC [45].

Similarly, the OIP cannot be directly observed but is commonly estimated at 1–2 years for CRC [46–50]. If a cancer is screen-detected, on average half of the OIP duration will have elapsed, whereas the entire OIP will have elapsed if the cancer is detected clinically through investigation of symptoms (Fig 1). Therefore, we formed separate strata for the look-back period for clinically-detected and screen-detected cases [44]. Cases were classified as screen-detected if a screening or surveillance endoscopy occurred within 3 months prior to the diagnosis date. Otherwise, the case was classified as clinically detected.

Our base-case analyses assumed an OIP of 1 year and PIDP of 5 years [46–49,51]. We assigned a look-back period encompassing the duration of the PIDP prior to the OIP (1–6 years prior to the reference date) for clinically-detected cases, and the PIDP prior to the OIP divided by 2 (0.5–5.5 years prior to the reference date) for screen-detected cases [44,52]. We performed sensitivity analyses with a 2-year estimated OIP duration and extending the look-back period to all available data.

### Classification of exposure

We defined three exposure categories: 1) no screening by lower endoscopy, 2) flexible sigmoidoscopy screening, and 3) colonoscopy screening. Because ascertainment of FOBT in claims data is poor [53], we did not consider this exposure. The no screening by lower endoscopy group included subjects without a screening/surveillance colonoscopy or sigmoidoscopy during the look-back period. The flexible sigmoidoscopy screening group included subjects whose first or only screening endoscopy in the look-back period was a flexible sigmoidoscopy and subjects with an initial screening flexible sigmoidoscopy followed by one or more surveillance colonoscopies. We assumed that any surveillance colonoscopies were performed to follow-up on abnormalities from the initial screening sigmoidoscopy. The colonoscopy screening group included subjects whose first or only screening/surveillance exam was a colonoscopy with no screening sigmoidoscopies and subjects with a surveillance sigmoidoscopy after any screening or surveillance colonoscopy. Cases with more complex exposure patterns (for example, both screening colonoscopy and sigmoidoscopy) and their matched controls were excluded due to ambiguity in attributing exposure. If a control had a longer exposure window than their matched case, we tabulated screening exams in him/her only within the case’s look-back period.

### Other covariates

We classified ecologic-level socioeconomic status using the median residential ZIP code income from U.S. Census data, and residential urbanicity using the Rural-Urban Commuting Area system [54]. We classified comorbidity with an adapted Charlson comorbidity index developed for use in SEER-Medicare data [55]. We identified family history of CRC using ICD9-CM codes V16.0 (family history—gastrointestinal neoplasm). Because a greater proportion of controls than cases had at least one claim with this code, we believed ascertainment of family history using these data was unreliable and did not include this variable in our analysis.

We classified endoscopist specialty by linking the Unique Physician Identification Number or National Provider Identification of the endoscopist on the procedure claim to the American Medical Association (AMA) Physician Masterfile [56,57]. If the identifiers could not be linked to the AMA Physician Masterfile, we assigned the specialty listed in the Medicare claim. Endoscopist-related variables were classified as unknown/missing for the <1% of procedures with multiple physician identifiers on the Medicare claims. Endoscopist specialty was categorized as gastroenterology, surgery, primary care, and other/unknown. Lastly, we determined endoscopists’ polyp detection rate by aggregating data across all their submitted colonoscopy claims in the 5% non-cancer claims. The codes for colon polyps are highly accurate in the Medicare claims, with a sensitivity of over 93% and specificity over 98% [58]. Polyp detection rate was calculated as the proportion of all colonoscopies with an ICD-9-CM code for a colorectal polyp (S1 Table). We did not estimate polyp detection rate for flexible sigmoidoscopy. Because pathology results were unavailable, we could not determine adenoma detection rate.

### Data analysis

All analyses were conducted using SAS version 9.4 (SAS Institute, Cary, North Carolina). We developed conditional logistic regression models to estimate the odds ratios (OR) and 95% confidence intervals (CI) for history of either screening colonoscopy or flexible sigmoidoscopy in cases compared to matched controls. Exposure was modeled in 3 categories as previously described. All models were adjusted for comorbidity, residential urbanicity, and median residential ZIP code income.

We performed secondary analyses stratified by sex, race, and cancer location. We also examined whether associations differed according to endoscopist characteristics. In these models, endoscopist-related variables were entered as dummy variables, and the reference group was no screening.

### Institutional review board approval

The study was approved by the Cancer Consortium Institutional Review Board of the Fred Hutchinson Cancer Research Center and the University of Washington. The SEER-Medicare data are a limited data set; personal identifiers (Social Security numbers, names, medical record numbers) are not included in the released data, but personal health information (treatment dates) are included. The Institutional Review Board waived the requirement for informed consent for use of this data.

## Results

**Fig 2:**
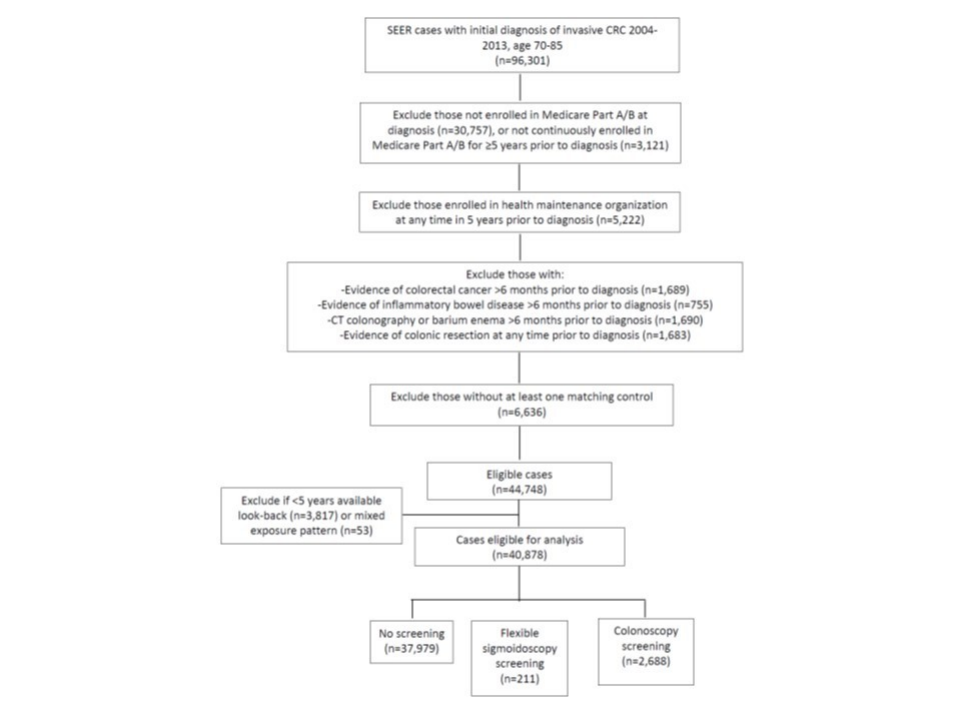
Selection of cases. CRC–colorectal cancer; SEER–Surveillance, Epidemiology, and End-Results.

We identified 96,301 potential subjects with invasive CRC diagnosed during 2004–2013. After applying exclusion criteria, 51,384 eligible cases remained. After excluding 6,636 cases without matching controls, 3,821 with less than 5 years available look-back, and 52 with a mixed exposure pattern, 40,875 cases were available for analysis (Fig 2). Only one matching control was available for 10,356 cases, and only 2 matching controls for 9,509 cases. The base-case analysis included 92,404 controls. Demographic characteristics of cases and controls were similar (Table 1). In the base-case analysis, 48.1% and 47.7% of CRCs were located in the proximal and distal colon, respectively (Table 2). Approximately 77% of CRCs were diagnosed at local/regional stage and 18.5% at distant stage. Seventeen percent of cases were screen-detected. Because of differences in required look-back time, the number of subjects varied in sensitivity analyses of the OIP and look-back period duration (S2 Table).

**Table 1:** Characteristics of colorectal cancer cases diagnosed during 2004–2013 and their matched controls, SEER-Medicare*.

	Cases N = 40,875	Controls N = 92,404
Age, years, at index date (n, %)		
70–74	11,888 (29.1)	31,007 (33.6)
75–79	14,392 (35.2)	32,859 (35.6)
80–85	14,595 (35.7)	28,538 (30.9)
Sex (n, %)		
Female	22,643 (55.4)	53,054 (57.4)
Race (n, %)		
White	34,555 (84.5)	77,747 (84.1)
African-American	3,326 (8.1)	7,274 (7.9)
Other/unknown	2,994 (7.3)	7,383 (8.0)
Median income, ZIP code of residence		
<$40,000	81,76 (20.0)	18,274 (19.8)
$40–49,999	7,926 (19.4)	18,674 (20.2)
$50–59,999	7,030 (17.2)	15,999 (17.3)
$60–79,999	8,980 (22.0)	20,232 (21.9)
>$80,000	7,231 (17.7)	17,004 (18.4)
Unknown	1,532 (3.7)	2,221 (2.4)
Rural-urban residence (n, %)		
Large metropolitan	21,187 (51.8)	45,613 (49.4)
Metropolitan	11,833 (28.9)	28,893 (31.3)
Urban	2,595 (6.3)	6,177 (6.7)
Less urban	4,252 (10.4)	9,231 (10.0)
Rural/unknown	1,008 (2.5)	2,299 (2.4)
Charlson comorbidity score (n, %)		
0	17,754 (43.4)	44,552 (48.2)
1	10,221 (25.0)	21,322 (23.1)
2+	11,120 (27.2)	20,858 (22.6)
Unknown	1,780 (4.4)	5,672 (6.1)
Classification of screening history (n, %)		
No screening	37,992 (92.9)	78,206 (84.6)
Flexible sigmoidoscopy screening	209 (0.5)	552 (0.6)
Colonoscopy screening	2,674 (6.5)	13,646 (14.8)

*SEER: Surveillance, Epidemiology, and End Results. Base-case analysis in which occult invasive period = 1 year and look-back period = 5 years.

**Table 2:** Characteristics of colorectal cancer cases diagnosed during 2004–2013, SEER-Medicare*.

	All Cases N = 40,875	Screening During the Pre-Invasive Detectable Phase		
		No Screening by Lower Endoscopy N = 37,992	Flexible Sigmoidoscopy Screening N = 209	Colonoscopy Screening N = 2,674
Year of diagnosis (n, %)				
2004–6	13,752 (33.6)	13,110 (34.5)	139 (66.5)	503 (18.8)
2007–9	12,509 (30.6)	11,624 (30.6)	49 (23.4)	836 (31.3)
2010–13	14,614 (35.8)	13,258 (34.9)	21 (10.0)	1,335 (49.9)
Site (n, %)				
Proximal	19,678 (48.1)	17,660 (46.5)	136 (65.1)	1,882 (70.4)
Distal	19,498 (47.7)	18,723 (49.3)	73 (34.9)	709 (26.5)
Unknown	1,699 (4.2)	1,609 (4.2)	--†	83 (3.1)
SEER Historic Stage A (n, %)				
Local	17,136 (41.9)	15,672 (41.3)	98 (46.9)	1,366 (51.1)
Regional	14,174 (34.7)	13,201 (34.7)	78 (37.3)	895 (33.5)
Distant	7,555 (18.5)	7,172 (18.9)	33 (15.8)	351 (13.1)
Unknown	2,010 (4.9)	1,947 (5.1)	--†	62 (2.3)
Method of detection (n, %)				
Screening or surveillance	6,795 (16.6)	5,693 (15.0)	43 (20.6)	1,059 (39.6)

*SEER: Surveillance, Epidemiology, and End Results. Base-case analysis in which occult invasive period = 1 year and look-back period = 5 years.

†Fewer than 11 cases were identified, and this cell was combined with cell above to adhere to SEER-Medicare data use policies.

Almost 93% of cases were classified in the no screening by lower endoscopy, 0.5% in the screening flexible sigmoidoscopy and 6.5% in the screening colonoscopy groups, compared to 84.6%, 0.6%, and 14.8% of controls, respectively (Table 1). There were minimal differences in subject characteristics or exposure classification for subjects in the base-case compared to the sensitivity analyses (S2 and S3 Tables).

### Overall effectiveness

Screening flexible sigmoidoscopy was associated with a 22% reduction in CRC incidence (OR 0.78, 95%CI 0.67, 0.92), and screening colonoscopy with a 59% reduction (OR 0.41, 95%CI 0.39, 0.43) (Table 3). Using an OIP of 1 year and all available years of look-back data, flexible sigmoidoscopy screening was associated with a 23% reduction in CRC incidence (OR 0.77; 95%CI 0.67, 0.88), and screening colonoscopy with a 58% reduction (OR 0.42; 95%CI 0.40, 0.43) (S5 Table). Estimates were similar using an OIP of 2 years (S4 Table).

**Table 3:** Association of screening/surveillance colonoscopy or flexible sigmoidoscopy with colorectal cancer incidence overall and stratified by selected variables, SEER-Medicare*.

	Number of cases	Flexible Sigmoidoscopy vs. No screening ADJUSTED ODDS RATIO† (95%CI)	Colonoscopy vs. No screening ADJUSTED ODDS RATIO† (95%CI)
Overall	40,875	0.78 (0.67, 0.92)	0.41 (0.39, 0.43)
Site of colon cancer			
Proximal	19,678	1.10 (0.89, 1.36)	0.62 (0.59, 0.66)
Distal	19,498	0.48 (0.37, 0.63)	0.22 (0.20, 0.24)
Unknown	1,699	1.31 (0.52, 3.32)	0.30 (0.23, 0.39)
Sex			
Male	18,232	0.69 (0.54, 0.88)	0.37 (0.34, 0.39)
Female	22,643	0.88 (0.70, 1.10)	0.45 (0.42, 0.48)
Race			
White	34,555	0.79 (0.66, 0.94)	0.42 (0.40, 0.44)
Black	3,326	0.90 (0.47, 1.72)	0.31 (0.26, 0.38)
Other/unknown	2,994	0.70 (0.37, 1.32)	0.36 (0.30, 0.45)

*SEER: Surveillance, Epidemiology, and End Results. Base-case analysis in which occult invasive period = 1 year and look-back period = 5 years.

†Adjusted for comorbidity, median income in ZIP code of residence, and rural-urban residence.

### Additional analyses

Flexible sigmoidoscopy was associated with a 52% reduction in distal CRC incidence (OR 0.48, 95%CI 0.37, 0.63), but with no reduction in proximal CRC incidence (OR 1.10, 95%CI 0.89, 1.36). Colonoscopy was associated with greater reductions in CRC incidence in the distal colon (OR 0.22, 95%CI 0.20, 0.24) than in the proximal colon (OR 0.62, 95%CI 0.59, 0.66) (Table 4). The reduction in CRC incidence associated with screening sigmoidoscopy or colonoscopy was somewhat greater in men than in women, but was similar in the different racial categories (Table 3).

**Table 4:** Endoscopist characteristics in relation to the magnitude of the association of CRC incidence and screening endoscopy history, SEER-Medicare*.

	Adjusted Odds Ratio† (95%CI)
Endoscopist specialty	
Colonoscopy by gastroenterologist	0.39 (0.37, 0.41)
Colonoscopy by surgeon	0.47 (0.43, 0.52)
Colonoscopy by primary care provider	0.46 (0.38, 0.56)
Colonoscopy by unknown specialty	0.33 (0.19, 0.56)
Sigmoidoscopy by gastroenterologist	0.68 (0.51, 0.90)
Sigmoidoscopy by surgeon	0.69 (0.36, 1.34)
Sigmoidoscopy by primary care provider	0.87 (0.70, 1.09)
Sigmoidoscopy by unknown specialty	0.76 (0.39, 1.48)
Polyp detection rate	
Colonoscopy by lowest quartile endoscopist	0.49 (0.45, 0.54)
Colonoscopy by second quartile endoscopist	0.44 (0.40, 0.48)
Colonoscopy by third quartile endoscopist	0.41 (0.38, 0.45)
Colonoscopy by highest quartile endoscopist	0.34 (0.31, 0.37)
Colonoscopy, unknown polyp detection rate endoscopist	0.44 (0.35, 0.57)
Sigmoidoscopy	0.78 (0.67, 0.92)

*SEER: Surveillance, Epidemiology, and End Results. Base-case analysis in which occult invasive period = 1 year and look-back period = 5 years.

†Adjusted for comorbid conditions, median income in ZIP code of residence, and rural-urban residence. Reference group is no screening.

Colonoscopy screening appeared to be similarly effective regardless of endoscopist specialty (Table 4). There was a suggestion that sigmoidoscopy performed by a gastroenterologist was associated with a greater reduction in CRC incidence (OR 0.68, 95%CI 0.51, 0.90) than when performed by a primary care provider (OR 0.87, 95%CI 0.70, 1.09). Because screening sigmoidoscopy by a surgeon was uncommon, confidence intervals for this estimate were wide.

Finally, we examined effectiveness of colonoscopy by quartile of endoscopist polyp detection (Table 4). Colonoscopy was associated with the greatest incidence reduction if performed by endoscopists in the highest polyp detection quartile (OR 0.34, 95%CI 0.31, 0.37) but was associated with reduced incidence even if performed by endoscopists in the lowest quartile (OR 0.49, 95%CI 0.45, 0.54). Results of additional sensitivity analyses with varying durations of the OIP or the look-back period were similar (S4 and S5 Tables).

## Discussion

In this case-control study, both screening flexible sigmoidoscopy and colonoscopy were associated with reductions in overall CRC incidence, although the magnitude of the reduction was greater for colonoscopy. Colonoscopy was associated with greater reductions in incidence of distal CRC compared to proximal CRC. Flexible sigmoidoscopy was not associated with any reduction in proximal CRC incidence. These findings were similar across different assumptions about the duration of the OIP and the PIDP.

Our results are consistent with randomized controlled trials of flexible sigmoidoscopy [9–12]. The United Kingdom flexible sigmoidoscopy screening trial found a 23–26% reduction in CRC incidence after a one-time exam, with the effect primarily in the distal colon [9,32]. The U.S. PLCO trial found a 21% reduction in CRC incidence after screening sigmoidoscopy, again with greater reductions in the distal than the proximal colon [10]. The Norwegian Colorectal Cancer Prevention Trial also found a 20% reduction in CRC incidence after one-time flexible sigmoidoscopy with or without FOBT [12]. Finally, the Italian SCORE trial found an 18% reduction in CRC incidence after one-time flexible sigmoidoscopy [11].

The observed reduction in overall CRC incidence associated with receipt of screening colonoscopy is similar in direction and magnitude to prior non-randomized studies [17,18]. A German case-control study found a 77% reduction in incidence of CRC associated with any colonoscopy in the prior 10 years, again with greater reductions seen in the distal colon [17]. However, this study included colonoscopies performed for any indication, potentially introducing a source of bias. An analysis of the Health Professionals Follow-Up Study and Nurses’ Health Study cohorts found a 43% reduction in overall CRC incidence after colonoscopy for any indication with polypectomy, and a 56% reduction after a negative colonoscopy [18]. Results were similar when restricting to endoscopy performed for screening indications. In addition, an 8-year follow-up of 70–74 year-old Medicare recipients observed a cumulative CRC incidence of 2.19 per 100 in persons undergoing a first screening colonoscopy and 2.62 per 100 in persons who did not undergo screening, implying a 16% reduction associated with receipt of screening [59]. This estimate of relative benefit is smaller than that suggested by others, likely because of inclusion of CRC cases that were diagnosed at colonoscopy which could not have been prevented by screening.

Strengths of our study include comparison of sigmoidoscopy and colonoscopy in the same underlying population eligible for screening and time period, and our attempt to avoid bias by including only endoscopies judged to have been performed for screening or surveillance. Because of the size of the study population, we were also able to do several sub-analyses. Our study also complements prior literature by analyzing endoscopist-level data, including specialty and polyp detection rate.

Limitations include potential inaccuracies in determining endoscopy indication. However, we used a validated algorithm for screening/surveillance vs. diagnostic procedures. In addition, for studies of screening and cancer incidence, only exams that occur during the PIDP are counted, and the need to ascertain indication is less critical than for studies of cancer mortality [44]. Because of the structure of the SEER-Medicare database, the length of the available look-back period was limited for some study subjects, and we could not account for screening that might have occurred prior to the available look-back period. Nevertheless, sensitivity analyses using varying estimates of the OIP and look-back periods had similar results. Cases diagnosed by sigmoidoscopy tended to occur earlier in the study period. If substantial technological advances occurred over the study period, estimates in the relative strength of association for the two procedures may be less accurate. We also studied an older population, ages 70–85 years, although screening is generally recommended to begin at age 50. These results therefore may not generalize to a younger population. Nevertheless, if screening is beneficial for reducing CRC incidence for up to 10 years, individuals up age 85 may benefit from screening taking place up to age 75, as is currently recommended. The magnitude of incidence reduction was similar to that observed in randomized controlled studies of screening sigmoidoscopy [9–12], but less than that seen in prior observational studies of screening colonoscopy [13,15,17,60]–all of which generally included younger subjects. The reasons for the differences in incidence reduction are unknown, but may relate to differences in tumor biology or aggressiveness in the different age groups studied. These differences may also be related to the observational nature of our study, which estimates effectiveness in clinical practice compared to estimates of efficacy seen in the randomized controlled studies.

Although combined flexible sigmoidoscopy and FOBT is a recommended screening regimen, we did not study this combination due to anticipated incomplete ascertainment of FOBT. We were unable to reliably ascertain family history of CRC, although family history still impacts the risk of CRC in the elderly [61]. It is possible that some incomplete colonoscopies were coded as flexible sigmoidoscopies. However, in these cases, the visualized portion of the colon would correspond to what would have been seen on sigmoidoscopy, lessening the possibility of bias in our results. Lastly, we were not able to directly examine endoscopists’ adenoma detection rates, but were limited to polyp detection rates. However, levels of polyp and adenoma detection are correlated [62–64]. Although we attempted to control for potentially confounding variables, there may be some residual confounding.

In summary, we found that both screening flexible sigmoidoscopy and colonoscopy were associated with reductions in CRC incidence. Our results suggest greater reductions in incidence associated with colonoscopy than with sigmoidoscopy overall, and particularly in the right colon. These results provide additional information to providers, patients, and policy-makers in making decisions or recommendations about CRC screening.

## Supporting information

S1 Table CPT, HCPCS, and ICD9 codes for colorectal procedures and diagnoses. (DOCX)

S2 Table Characteristics of colorectal cancer cases diagnosed during 2004–2013 and their matched controls in secondary analyses, SEER-Medicare *. (DOCX)

S3 Table Characteristics of colorectal cancers diagnosed during 2004–2013 in secondary analyses, SEER-Medicare*. (DOCX)

S4 Table Association of screening colonoscopy or flexible sigmoidoscopy with colorectal cancer incidence in secondary analyses, SEER-Medicare*. (DOCX)

S5 Table Endoscopist characteristics in relation to the magnitude of the association of CRC incidence and screening endoscopy history, SEER-Medicare*. (DOCX)
