# Supplementary material for: Screening colonoscopy and flexible sigmoidoscopy for reduction of colorectal cancer incidence: A case-control study
Source: PLoS One. 2019 Dec 5;14(12):e0226027. doi: 10.1371/journal.pone.0226027 (PMC6894764; doi:10.1371/journal.pone.0226027)
Supplement: S1 Table — (DOCX) [file pone.0226027.s001.docx]

**S1 Table. CPT, HCPCS, and ICD9 codes for colorectal procedures and diagnoses**

|  | **CPT, HCPCS, ICD9-P, or ICD-9-CM Codes** |
| --- | --- |
| Colonoscopy | **CPT:** 45378, 45380, 45381, 45382, 45383, 45384, 45485  **HCPCS:** G0105, G0121  **ICD9-P:** 45.23, 45.25, 45.27, 45.41, 45.42, 45.43, 48.24, 48.36 |
| Flexible sigmoidoscopy | **CPT:** 45330, 45331, 45333, 45334, 45335, 45338, 45339  **HCPCS:** G0104  **ICD9-P:** 45.24 |
| Colorectal polyp | **ICD-9-CM:** 569.0, 211.3, 211.4 |
