# Supplementary material for: Screening colonoscopy and flexible sigmoidoscopy for reduction of colorectal cancer incidence: A case-control study
Source: PLoS One. 2019 Dec 5;14(12):e0226027. doi: 10.1371/journal.pone.0226027 (PMC6894764; doi:10.1371/journal.pone.0226027)
Supplement: S2 Table — (DOCX) [file pone.0226027.s002.docx]

**S2 Table. Characteristics of colorectal cancer cases diagnosed during 2004-2013 and their matched controls in secondary analyses, SEER-Medicare***

|  | | |
| --- | --- | --- |
| **Occult invasive period = 1 year and look-back period = all available years†** | | |
|  | Cases  N= 44,647 | Controls  N= 102,524 |
| Age, years, at index date (n, %) |  |  |
| 70-74 | 14,904 (33.4) | 39,304 (38.3) |
| 75-79 | 14,825 (33.2) | 33,917 (33.1) |
| 80-85 | 14,918 (33.4) | 29,303 (28.6) |
| Sex (n, %) |  |  |
| Female | 24,463 (54.8) | 58,048 (56.6) |
| Race (n, %) |  |  |
| White | 37,560 (84.1) | 85,864 (83.7) |
| African-American | 3,759 (8.4) | 8,376 (8.2) |
| Other/unknown | 3,328 (7.5) | 8,284 (7.1) |
| Median income, ZIP code of residence |  |  |
| <$40,000 | 8,942 (20.0) | 20,454 (20.0) |
| $40-49,999 | 8,643 (19.4) | 20,728 (20.2) |
| $50-59,999 | 7,654 (17.10 | 17,729 (17.3) |
| $60-79,999 | 9,824 (22.0) | 22,313 (21.8) |
| >$80,000 | 7,895 (17.7) | 18,802 (18.3) |
| Unknown | 1,689 (3.8) | 2,498 (2.4) |
| Rural-urban residence (n, %) |  |  |
| Large metropolitan | 23,177 (51.9) | 50,644 (49.4) |
| Metropolitan | 12,945 (29.0) | 32,012 (31.2) |
| Urban | 2,834 (6.3) | 6,816 (6.6) |
| Less urban | 4,607 (10.3) | 10,274 (10.0) |
| Rural/unknown | 1,084 (2.4) | 2,778 (2.3) |
| Charlson comorbidity score (n, %) |  |  |
| 0 | 19,483 (43.6) | 49,564 (48.3) |
| 1 | 11,115 (24.9) | 23,472 (22.9) |
| 2+ | 12,021 (26.9) | 22,996 (22.4) |
| Unknown | 2,028 (4.5) | 6,492 (6.3) |
| Classification of screening history (n, %) |  |  |
| No screening | 41,063 (92.0) | 85,265 (83.2) |
| Flexible sigmoidoscopy screening | 300 (0.7) | 807 (0.8) |
| Colonoscopy screening | 3,284 (7.4) | 16,452 (16.0) |
|  |  |  |
| **Occult invasive period = 2 years and look-back period = 5 years** | | |
|  | Cases  N= 20,917 | Controls  N= 46,702 |
| Age, years, at index date (n, %) |  |  |
| 70-74 | 5,309 (25.4) | 13,822 (29.6) |
| 75-79 | 7,955 (38.0) | 18,133 (38.8) |
| 80-85 | 7,653 (36.6) | 14,747 (31.6) |
| Sex (n, %) |  |  |
| Female | 11,726 (56.1) | 27,238 (58.4) |
| Race (n, %) |  |  |
| White | 17,857 (85.4) | 39,701 (85.0) |
| African-American | 1,584 (7.6) | 3,397 (7.3) |
| Other/unknown | 1,476 (7.1) | 3,604 (7.7) |
| Median income, ZIP code of residence |  |  |
| <$40,000 | 3,976 (19.0) | 8,932 (19.1) |
| $40-49,999 | 4,007 (19.2) | 9,373 (20.1) |
| $50-59,999 | 3,626 (17.3) | 8,248 (17.7) |
| $60-79,999 | 4,696 (22.5) | 10,419 (22.3) |
| >$80,000 | 3,832 (18.3) | 8,610 (18.4) |
| Unknown | 780 (3.7) | 1,120 (2.4) |
| Rural-urban residence (n, %) |  |  |
| Large metropolitan | 10,870 (52.0) | 22,988 (49.2) |
| Metropolitan | 6,076 (29.0) | 14,759 (31.6) |
| Urban | 1,300 (6.2) | 3,119 (6.7) |
| Less urban | 2,176 (10.4) | 4,591 (9.8) |
| Rural/unknown | 494 (2.4) | 1,118 (2.4) |
| Charlson comorbidity score (n, %) |  |  |
| 0 | 9,282 (44.4) | 22,427 (48.0) |
| 1 | 5,331 (25.5) | 10,808 (23.1) |
| 2+ | 5,554 (26.6) | 10,662 (22.8) |
| Unknown | 750 (3.6) | 2,805 (6.0) |
| Classification of screening history (n, %) |  |  |
| No screening | 18,949 (90.6) | 39,329 (84.2) |
| Flexible sigmoidoscopy screening | 134 (0.6) | 340 (0.7) |
| Colonoscopy screening | 1,834 (8.8) | 7,033 (15.1) |
|  | | |
| **Occult invasive period = 2 years and look-back period = all available years††** | | |
|  | Cases  N= 44,675 | Controls  N= 102,685 |
| Age, years, at index date (n, %) |  |  |
| 70-74 | 14,909 (33.4) | 39,371 (38.3) |
| 75-79 | 14,837 (33.2) | 33,972 (33.1) |
| 80-85 | 14,929 (33.4) | 29,342 (28.6) |
| Sex (n, %) |  |  |
| Female | 24,476 (54.8) | 58,125 (56.6) |
| Race (n, %) |  |  |
| White | 37,586 (84.1) | 86,005 (83.8) |
| African-American | 3,761 (8.4) | 8,392 (8.2) |
| Other/unknown | 3,328 (7.4) | 8,288 (8.1) |
| Median income, ZIP code of residence |  |  |
| <$40,000 | 8,948 (20.0) | 20,481 (19.9) |
| $40-49,999 | 8,646 (19.4) | 20,763 (21.8) |
| $50-59,999 | 7,656 (17.1) | 17,761 (17.3) |
| $60-79,999 | 9,833 (22.0) | 22,345 (21.8) |
| >$80,000 | 7,903 (17.7) | 18,831 (18.3) |
| Unknown | 1,689 (3.8) | 2,504 (2.4) |
| Rural-urban residence (n, %) |  |  |
| Large metropolitan | 23,196 (51.9) | 50,720 (49.4) |
| Metropolitan | 12,949 (29.0) | 32,058 (31.2) |
| Urban | 2,835 (6.3) | 6,829 (6.7) |
| Less urban | 4,609 (10.3) | 10,296 (10.0) |
| Rural/unknown | 1,086 (2.4) | 2,782 (2.7) |
| Charlson comorbidity score (n, %) |  |  |
| 0 | 19,493 (43.6) | 49,648 (48.4) |
| 1 | 11,124 (24.9) | 23,523 (22.9) |
| 2+ | 12,029 (26.9) | 23,021 (22.4) |
| Unknown | 2,029 (4.5) | 6,493 (6.3) |
| Classification of screening history (n, %) |  |  |
| No screening | 41,342 (92.5) | 87,793 (85.5) |
| Flexible sigmoidoscopy screening | 288 (0.6) | 799 (0.8) |
| Colonoscopy screening | 3,045 (6.8) | 14,093 (13.7) |

*SEER: Surveillance, Epidemiology, and End Results.

†Median look-back period 69 months, mean look-back period 69.5 months, interquartile range 63-76 months

†† Median look-back period 58 months, mean look-back period 58.4 months, interquartile range 51-65 months
