# Supplementary material for: Screening colonoscopy and flexible sigmoidoscopy for reduction of colorectal cancer incidence: A case-control study
Source: PLoS One. 2019 Dec 5;14(12):e0226027. doi: 10.1371/journal.pone.0226027 (PMC6894764; doi:10.1371/journal.pone.0226027)
Supplement: S3 Table — (DOCX) [file pone.0226027.s003.docx]

**S3 Table. Characteristics of colorectal cancer cases diagnosed during 2004-2013 in secondary analyses, SEER-Medicare***

|  |  | |  | |  | |  | |
| --- | --- | --- | --- | --- | --- | --- | --- | --- |
| **Occult invasive period = 1 year and look-back period = all available years** | | | | | | | | |
|  | | All Cases  N=44,647 | | No Screening  N=41,063 | | Flexible Sigmoidoscopy Screening  N=300 | | Colonoscopy Screening  N=3,284 |
| Year of diagnosis (n, %) | |  | |  | |  | |  |
| 2004-6 | | 15,107 (33.8) | | 14,336 (34.9) | | 181 (60.3) | | 590 (18.0) |
| 2007-2009 | | 13,809 (30.9) | | 12,689 (30.9) | | 94 (31.3) | | 1,026 (31.2) |
| 2010-13 | | 15,731 (35.2) | | 14,038 (34.2) | | 25 (8.3) | | 1,668 (50.8) |
| Site (n, %) | |  | |  | |  | |  |
| Proximal | | 21,350 (47.8) | | 18,889 (46.0) | | 190 (63.3) | | 2,271 (69.2) |
| Distal | | 21,472 (48.1) | | 20,469 (49.8) | | 110 (36.7) | | 904 (27.5) |
| Unknown | | 1,825 (4.1) | | 1,705 (4.2) | | --† | | 109 (3.3) |
| SEER Historic Stage A (n, %) | |  | |  | |  | |  |
| Local | | 18,671 (41.8) | | 16,845 (41.0) | | 140 (46.7) | | 1,686 (51.3) |
| Regional | | 15,540 (34.8) | | 14,342 (34.9) | | 113 (37.7) | | 1,085 (33.0) |
| Distant | | 8,314 (18.6) | | 7,832 (19.1) | | 47 (15.6) | | 439 (13.4) |
| Unknown | | 2,122 (4.8) | | 2,044 (5.0) | | --† | | 74 (2.3) |
| Method of detection (n, %) | |  | |  | |  | |  |
| Screening or surveillance | | 7,133 (16.0) | | 5,760 (14.0) | | 68 (22.7) | | 1,305 (39.7) |
|  | |  | |  | |  | |  |
| **Occult invasive period = 2 years and look-back period = 5 years** | | | | | | | | |
|  | | All Cases  N=20,917 | | No Screening  N=18,949 | | Flexible Sigmoidoscopy Screening  N=134 | | Colonoscopy Screening  N=1,834 |
| Year of diagnosis (n, %) | |  | |  | |  | |  |
| 2004-6 | | 5,117 (24.5) | | 4,766 (25.2) | | 71 (53.0) | | 280 (15.3) |
| 2007-2009 | | 7,930 (37.9) | | 7,270 (38.4) | | 63 (47.0) | | 607 (33.1) |
| 2010-13 | | 7.870 (37.6) | | 6,913 (36.5) | | --† | | 947 (51.6) |
| Site (n, %) | |  | |  | |  | |  |
| Proximal | | 10,444 (49.9) | | 9,069 (47.9) | | 86 (64.2) | | 1,289 (70.3) |
| Distal | | 9,656 (46.2) | | 9,116 (48.1) | | 48 (35.8) | | 495 (27.0) |
| Unknown | | 817 (3.9) | | 764 (4.0) | | --† | | 50 (2.7) |
| SEER Historic Stage A (n, %) | |  | |  | |  | |  |
| Local | | 9,205 (44.0) | | 8,114 (42.8) | | 61 (45.5) | | 1,030 (56.2) |
| Regional | | 7,135 (34.1) | | 6,496 (34.3) | | 55 (41.0) | | 584 (31.8) |
| Distant | | 3,601 (17.2) | | 3,401 (17.9) | | 18 (13.5) | | 183 (10.0) |
| Unknown | | 976 (4.7) | | 938 (5.0) | | --† | | 37 (2.0) |
| Method of detection (n, %) | |  | |  | |  | |  |
| Screening or surveillance | | 6,391 (30.6) | | 5,298 (28.0) | | 50 (37.3) | | 1,043 (56.9) |
|  | | | | | | | | |
| **Occult invasive period = 2 years and look-back period = all available years** | | | | | | | | |
|  | | All cases  N=44,675 | | No screening  N=41,342 | | Flexible sigmoidoscopy screening  N=288 | | Colonoscopy screening  N=3,045 |
| Year of diagnosis (n, %) | |  | |  | |  | |  |
| 2004-6 | | 15,113 (33.8) | | 14,414 (34.9) | | 172 (59.7) | | 527 (17.3) |
| 2007-2009 | | 13,820 (30.9) | | 12,786 (30.9) | | 90 (31.3) | | 944 (31.0) |
| 2010-13 | | 15,742 (35.2) | | 14,142 (34.2) | | 26 (9.0) | | 1,574 (51.7) |
| Site (n, %) | |  | |  | |  | |  |
| Proximal | | 21,365 (47.8) | | 19,078 (46.1) | | 182 (63.2) | | 2,105 (69.1) |
| Distal | | 21,483 (48.1) | | 20,545 (49.7) | | 95 (33.0) | | 843 (27.7) |
| Unknown | | 1,827 (4.1) | | 1,719 (4.2) | | 11 (3.8) | | 97 (3.2) |
| SEER Historic Stage A (n, %) | |  | |  | |  | |  |
| Local | | 18,688 (41.8) | | 16,968 (41.0) | | 139 (48.3) | | 1,581 (51.9) |
| Regional | | 15,547 (34.8) | | 14,430 (34.9) | | 105 (36.5) | | 1,012 (33.2) |
| Distant | | 8,318 (18.6) | | 7,892 (19.1) | | 44 (15.3) | | 386 (12.7) |
| Unknown | | 2,122 (4.7) | | 2,052 (5.0) | | --† | | 66 (2.2) |
| Method of detection (n, %) | |  | |  | |  | |  |
| Screening or surveillance | | 7,138 (16.0) | | 5,804 (14.0) | | 69 (24.0) | | 1,265 (41.5) |

* SEER: Surveillance, Epidemiology, and End Results.

†Fewer than 11 cases were identified, and this cell was combined with cell immediately above
