# Supplementary material for: Screening colonoscopy and flexible sigmoidoscopy for reduction of colorectal cancer incidence: A case-control study
Source: PLoS One. 2019 Dec 5;14(12):e0226027. doi: 10.1371/journal.pone.0226027 (PMC6894764; doi:10.1371/journal.pone.0226027)
Supplement: S4 Table — (DOCX) [file pone.0226027.s004.docx]

**S4 Table. Association of screening colonoscopy or flexible sigmoidoscopy with colorectal cancer incidence in secondary analyses, SEER-Medicare***

|  | | |
| --- | --- | --- |
| **Occult invasive period = 1 year and look-back period = all available years** | | |
|  | Flexible sigmoidoscopy,  vs. No screening  Adjusted odds ratio† (95%CI) | Colonoscopy,  vs. No screening  Adjusted odds ratio† (95% CI) |
| Overall | 0.77 (0.67, 0.88) | 0.42 (0.40, 0.43) |
| Site of colon cancer |  |  |
| Proximal | 1.04 (0.87, 1.24) | 0.62 (0.59, 0.66) |
| Distal | 0.49 (0.39, 0.62) | 0.23 (0.21, 0.25) |
| Unknown | 1.33 (0.63, 2.82) | 0.34 (0.28, 0.43) |
| Sex |  |  |
| Male | 0.74 (0.61, 0.90) | 0.36 (0.34, 0.39) |
| Female | 0.79 (0.66, 0.96) | 0.47 (0.44, 0.49) |
| Race |  |  |
| White | 0.77 (0.67, 0.89) | 0.43 (0.41, 0.45) |
| Black | 0.69 (0.39, 1.21) | 0.34 (0.28, 0.40) |
| Other/unknown | 0.81 (0.48, 1.37) | 0.35 (0.29, 0.43) |
|  |  |  |
| **Occult invasive period = 2 years and look-back period = 5 years** | | |
|  | Flexible sigmoidoscopy,  vs. No screening  Adjusted odds ratio† (95% CI) | Colonoscopy,  vs. No screening  Adjusted odds ratio† (95% CI) |
| Overall | 0.80 (0.65, 0.98) | 0.55 (0.52, 0.58) |
| Site of colon cancer |  |  |
| Proximal | 1.09 (0.83, 1.43) | 0.80 (0.74, 0.86) |
| Distal | 0.53 (0.38, 0.74) | 0.31 (0.28, 0.34) |
| Unknown | 0.90 (0.23, 3.49) | 0.37 (0.26, 0.51) |
| Sex |  |  |
| Male | 0.88 (0.65, 1.18) | 0.49 (0.45, 0.54) |
| Female | 0.74 (0.55, 0.98) | 0.59 (0.55, 0.64) |
| Race |  |  |
| White | 0.80 (0.64, 1.00) | 0.56 (0.53, 0.59) |
| Black | 0.68 (0.30, 1.54) | 0.44 (0.34, 0.56) |
| Other/unknown | 0.92 (0.40, 2.12) | 0.48 (0.37, 0.62) |
|  |  |  |
| **Occult invasive period = 2 years and look-back period = all available years** | | |
|  | Flexible sigmoidoscopy,  vs. No screening  Adjusted odds ratio† (95%CI) | Colonoscopy,  vs. No screening  Adjusted odds ratio† (95%CI) |
| Overall | 0.76 (0.66, 0.87) | 0.46 (0.46, 0.48) |
| Site of colon cancer |  |  |
| Proximal | 1.03 (0.85, 1.23) | 0.68 (0.65, 0.72) |
| Distal | 0.49 (0.39, 0.62) | 0.26 (0.24, 0.28) |
| Unknown | 1.16 (0.55, 2.41) | 0.37 (0.29, 0.46) |
| Sex |  |  |
| Male | 0.73 (0.60, 0.89) | 0.41 (0.38, 0.43) |
| Female | 0.78 (0.65, 0.95) | 0.51 (0.49, 0.54) |
| Race |  |  |
| White | 0.76 (0.65, 0.88) | 0.47 (0.45, 0.49) |
| Black | 0.66 (0.37, 1.16) | 0.38 (0.31, 0.45) |
| Other/unknown | 0.89 (0.52, 1.52) | 0.41 (0.33, 0.49) |

*SEER: Surveillance, Epidemiology, and End Results

†Adjusted for comorbidity, median income in ZIP code of residence, and rural-urban residence.
