# Supplementary material for: Screening colonoscopy and flexible sigmoidoscopy for reduction of colorectal cancer incidence: A case-control study
Source: PLoS One. 2019 Dec 5;14(12):e0226027. doi: 10.1371/journal.pone.0226027 (PMC6894764; doi:10.1371/journal.pone.0226027)
Supplement: S5 Table — (DOCX) [file pone.0226027.s005.docx]

**S5 Table. Endoscopist characteristics in relation to the magnitude of the association of CRC incidence and screening endoscopy history, SEER-Medicare***

|  | |
| --- | --- |
| **Occult invasive period = 1 year and look-back period = all available years** | |
|  | Adjusted odds ratio† (95%CI) |
| Endoscopist specialty |  |
| Colonoscopy by gastroenterologist | 0.40 (0.38, 0.42) |
| Colonoscopy by surgeon | 0.48 (0.44, 0.52) |
| Colonoscopy by primary care provider | 0.48 (0.41, 0.57) |
| Colonoscopy by unknown specialty | 0.40 (0.25, 0.62) |
| Sigmoidoscopy by gastroenterologist | 0.64 (0.50, 0.81) |
| Sigmoidoscopy by surgeon | 0.85 (0.51, 1.42) |
| Sigmoidoscopy by primary care provider | 0.85 (0.71, 1.02) |
| Sigmoidoscopy by unknown specialty | 0.75 (0.43, 1.31) |
| Polyp detection rate |  |
| Colonoscopy by lowest quartile endoscopist | 0.50 (0.46, 0.55) |
| Colonoscopy by second quartile endoscopist | 0.45 (0.42, 0.49) |
| Colonoscopy by third quartile endoscopist | 0.41 (0.38, 0.44) |
| Colonoscopy by highest quartile endoscopist | 0.34 (0.32, 0.37) |
| Colonoscopy, unknown polyp detection rate | 0.45 (0.36, 0.57) |
| Sigmoidoscopy | 0.77 (0.67, 0.88) |
|  | |
| **Occult invasive period = 2 years and look-back period = 5 years** | |
|  | Adjusted odds ratio† (95%CI) |
| Endoscopist specialty |  |
| Colonoscopy by gastroenterologist | 0.52 (0.49, 0.56) |
| Colonoscopy by surgeon | 0.62 (0.55, 0.70) |
| Colonoscopy by primary care provider | 0.63 (0.49, 0.80) |
| Colonoscopy by unknown specialty | 0.43 (0.22, 0.86) |
| Sigmoidoscopy by gastroenterologist | 0.63 (0.44, 0.90) |
| Sigmoidoscopy by surgeon | 0.87 (0.42, 1.87) |
| Sigmoidoscopy by primary care provider | 0.95 (0.71, 1.26) |
| Sigmoidoscopy by unknown specialty | 0.63 (0.27, 1.49) |
| Polyp detection rate |  |
| Colonoscopy by lowest quartile endoscopist | 0.64 (0.57, 0.72) |
| Colonoscopy by second quartile endoscopist | 0.59 (0.53, 0.66) |
| Colonoscopy by third quartile endoscopist | 0.53 (0.47, 0.59) |
| Colonoscopy by highest quartile endoscopist | 0.47 (0.42, 0.52) |
| Colonoscopy, unknown polyp detection rate endoscopist | 0.58 (0.43, 0.78) |
| Sigmoidoscopy | 0.80 (0.65, 0.98) |
|  | |
| **Occult invasive period = 2 years and look-back period = all available years** | |
|  | Adjusted odds ratio† (95%CI) |
| Endoscopist specialty |  |
| Colonoscopy by gastroenterologist | 0.45 (0.43, 0.47) |
| Colonoscopy by surgeon | 0.50 (0.46, 0.55) |
| Colonoscopy by primary care provider | 0.54 (0.45, 0.64) |
| Colonoscopy by unknown specialty | 0.43 (0.27, 0.68) |
| Sigmoidoscopy by gastroenterologist | 0.64 (0.51, 0.82) |
| Sigmoidoscopy by surgeon | 0.83 (0.49, 1.39) |
| Sigmoidoscopy by primary care provider | 0.84 (0.70, 1.02) |
| Sigmoidoscopy by unknown specialty | 0.64 (0.36, 1.14) |
| Polyp detection rate |  |
| Colonoscopy by lowest quartile endoscopist | 0.54 (0.50, 0.59) |
| Colonoscopy by second quartile endoscopist | 0.50 (0.46, 0.55) |
| Colonoscopy by third quartile endoscopist | 0.45 (0.42, 0.49) |
| Colonoscopy by highest quartile endoscopist | 0.39 (0.36, 0.42) |
| Colonoscopy, unknown polyp detection rate endoscopist | 0.49 (0.39, 0.61) |
| Sigmoidoscopy | 0.76 (0.66, 0.87) |

*SEER: Surveillance, Epidemiology, and End Results

† Adjusted for comorbid conditions, median income in ZIP code of residence, and rural-urban residence. Reference group is no screening.
